# The sonographic Murphy sign: does analgesia matter?

**DOI:** 10.1007/s10140-025-02387-6

**Published:** 2025-09-24

**Authors:** Elianna L. Goldstein, Karina R. Marcelo, William R. Harjes, Jonathan R. Wood, Yang-En Kao

**Affiliations:** 1 Department of Radiology, California Advanced Imaging Medical Associates, San Francisco, CA USA; 2 Department of Radiology, Tripler Army Medical Center, Honolulu, HI USA; 3 Department of Radiology, Alaska Native Medical Center, Anchorage, AK USA; 4 Department of Radiology, Intermountain Primary Children’s Hospital, Salt Lake City, UT USA

**Keywords:** Sonographic Murphy sign, Analgesia, Opioids, Cholecystitis

## Abstract

**Purpose:**

Controversy exists regarding analgesia premedication prior to right upper quadrant ultrasound (RUQUS) in the setting of abdominal pain when evaluating for acute cholecystitis (AC). The purpose of this study was to examine the effect of opioid and non-opioid analgesia (OA and NOA, respectively) on the sonographic Murphy sign (maximal tenderness when an ultrasound transducer probe is pressed over the visualized gallbladder) and the radiologic accuracy of diagnosing AC.

**Methods:**

A retrospective cohort chart review analyzed 686 adult patients in two groups and the effect on diagnosis of AC in the emergency department: those who received OA versus control and another group comparing NOA versus control.

**Results:**

OA resulted in an increased rate of indeterminate sonographic Murphy sign and diagnoses in the treatment group compared to control (7.9% vs. 3.0%, respectively). This resulted in 24 cases of radiology-missed AC. However, there was no statistically significant difference in false-negative AC diagnosis between the NOA group compared to control (4.6% vs. 3.7%, respectively). Patients receiving OA within 30 minutes of their RUQUS examination were more likely to be given a false-negative diagnosis compared to control (8.5% vs 3.0%, respectively). Even morphine-equivalent doses <4mg were associated with increased false-negatives compared to control (8.0% vs 3.0%, respectively).

**Conclusions:**

Clinicians should consider delaying OA until after the RUQUS or consider delaying the study at least 30 minutes after the administration of OA due to increased risk of false-negative results. Additionally, our results suggest that administration of NOA is a viable alternative analgesic option for many patients without sacrificing diagnostic accuracy.

**Graphical Abstract:**

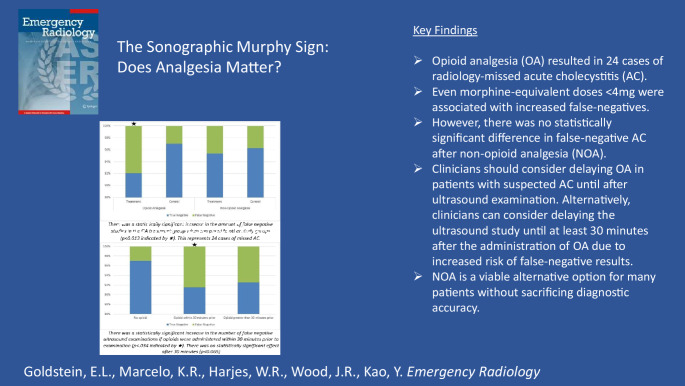

## Introduction

It is estimated that 10–15% of adults in developed countries have gallstones, and gallstone related disease is the most common cause of gastrointestinal related inpatient admissions [1, 2]. The prevalence of acute cholecystitis (AC) in the Western world is 5% with more than 700,000 cholecystectomies performed yearly in the United States [3, 4]. When a patient presents with a chief complaint of right upper quadrant (RUQ) pain, emergency physicians use a combination of patient history, physical examination findings, laboratory tests, and imaging to determine a diagnosis. Unfortunately, no single laboratory test, physical exam or imaging finding can reliably rule in or rule out AC [5]. In addition, it has been shown that approximately one-third of patients with suspected AC have a different diagnosis [6].

The right upper quadrant ultrasound (RUQUS) is a very sensitive tool to quickly eliminate acute biliary disease in most patients. In fact, the sensitivity of ultrasound has been found to be far superior to that of CT, 83% vs. 39% respectively, with similar specificities, 95% vs. 93% respectively [7]. Ultrasound findings of AC include gallstones, gallbladder wall thickening, an enlarged gallbladder, pericholecystic fluid, and the sonographic Murphy sign (SMS). The presence of any one of these findings is not specific for AC, however, the combination of gallstones and a positive SMS has been shown to have a 92% positive predictive value [8].

The SMS is defined as maximal tenderness elicited when the transducer probe is pressed over a sonographically localized gallbladder [9]. Unfortunately, the SMS is operator dependent and will likely vary depending on the experience of the sonographer. In addition, controversy exists regarding the diagnostic accuracy of the SMS when patients are given pain control medications prior to their RUQUS.

In 2017, the American College of Emergency Physicians put forth a policy statement on the optimal treatment of acute pain in patients presenting to the emergency department (ED). According to the statement, management of acute pain should begin first with a non-opioid medication when able. For example, non-steroidal anti-inflammatory drugs (NSAIDs) or acetaminophen should be considered first for treatment of acute pain. Other options include nerve blocks, sub-dissociative doses of ketamine, and lidocaine [10]. There are advantages to treating acute pain with non-opioid analgesia (NOA) versus opioid medications. One study found that patients treated with opioid analgesia (OA) in the ED had a statistically significant higher return rate to the ED (22.0%) within 30 days when compared to those treated with acetaminophen and/or NSAIDs (14.7%; *p* < 0.001) [11]. Therefore, it is important to consider the potential implications of these non-opioid medications on the SMS and the diagnosis of AC.

In practice, ED physicians often provide pain medication to patients presenting with undifferentiated abdominal pain, which may mask symptoms or alter abdominal exams to such a degree that critical diagnoses can be obscured. There are contradictory findings with regard to the SMS and the impact of premedication.

There is an absence of diagnostic radiology-based research on the impact of premedication on the SMS. We performed a retrospective analysis to determine whether analgesia, opioid or non-opioid, alters the reliability of the SMS in evaluating acute gallbladder pathology.

## Materials and methods

### Study design

This study consisted of a retrospective cohort chart review to test the null hypotheses that there is no significant difference in the assessment of the SMS in two sets of patients: those who have and have not received prior OA and a second group of patients who have or have not received NOA. Additionally, the sensitivity and specificity of the SMS for AC is similar in patients who have received prior OA or NOA versus their respective control groups. The study protocol was approved by the appropriate human subjects regulatory authority. Investigators adhered to the policies for protection of human subjects as prescribed in 45 Code of Federal Regulation 46.

### Setting and population

**Fig. 1:**
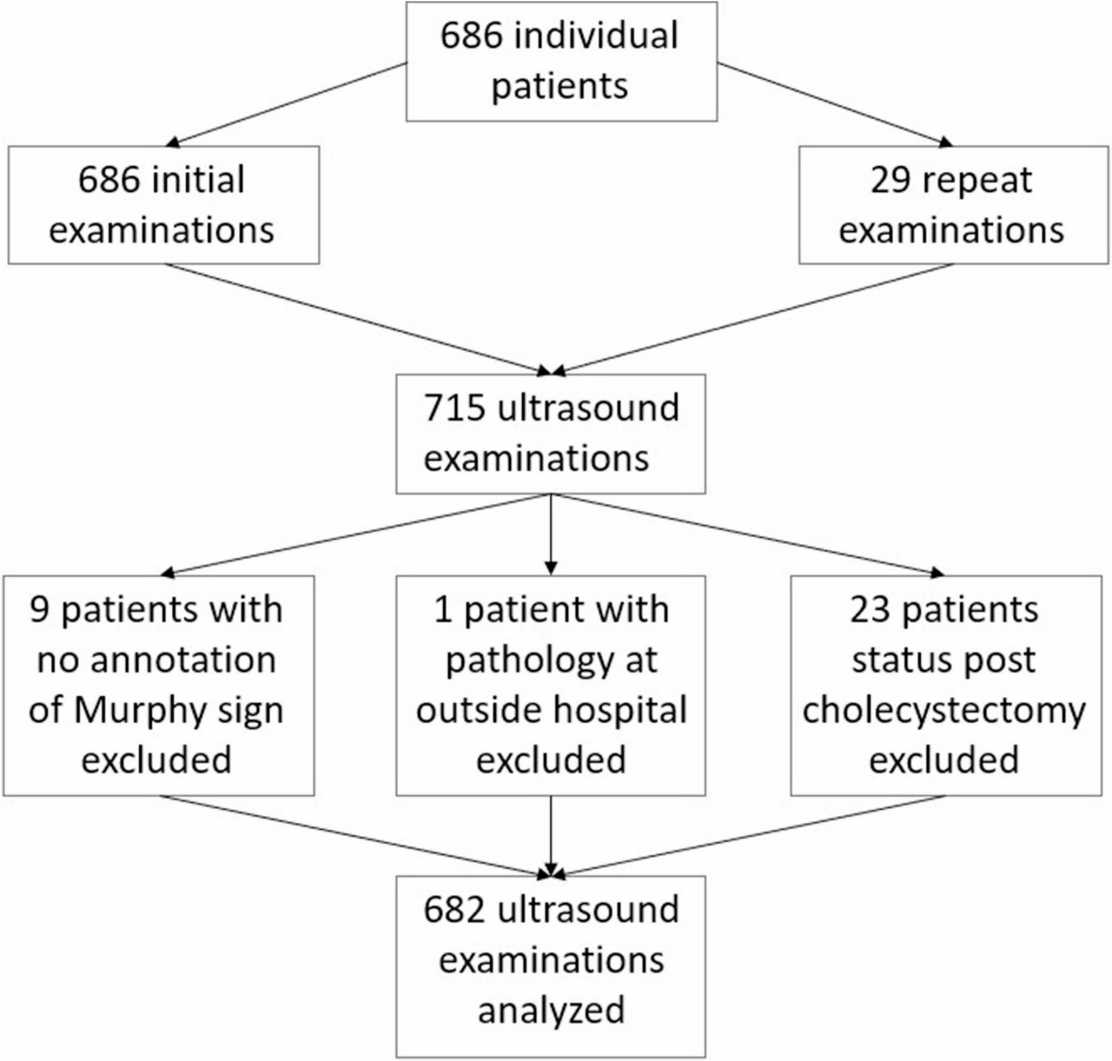
The breakdown of the 682 examinations included in the study

The study was conducted at a diagnostic radiology residency program with a Level II, tertiary care emergency department. The attending radiology staff are board-certified or eligible and the emergency staff are board-certified. Ultrasound examinations were performed by experienced technologists all of whom had at least 7 years of experience. The research subjects consisted of 686 adult patients who presented to the emergency department with unspecified abdominal pain and had either a RUQUS or gallbladder ultrasound during the study period from January 1, 2018 to September 1, 2019. Twenty-nine of these ultrasound exams were repeat exams from patients who had been previously scanned at least once during the study period for a total of 715 ultrasound exams for 686 individual adult patients. There were 23 patients scanned who had no gallbladder at the time of the exam, nine patients who had no annotation of present or absent SMS, and one patient who had pathology reported at an outside hospital; these patients were therefore excluded. A total of 682 ultrasound exams were analyzed (Fig. 1). The average age of patients at the time of their RUQUS was 38.5 years. About two-thirds of the patients (65.8%) were female and the rest (34.2%) were male. The racial/ethnic composition of the study participants were as follows: Caucasian 43.1%, African American/Black 13.5%, Asian/Pacific Islander 16.3%, Native American 0.4%, other 19%, and unknown 7.6% (Table 1).

**Table 1:** The baseline characteristics of the different study groups

	Opioid analgesia	Opioid control	Non-opioid analgesia	Non-opioid control
Racial background (number of patients)				
Caucasian	67	225	102	190
African American/Black	19	73	36	56
Asian/Pacific Islander	27	84	35	76
Native American	0	2	0	3
Other	35	95	50	80
Unknown	9	43	24	28
Gender (number of patients)				
Male	50	183	75	158
Female	108	341	173	276
Average age				
Years	38.2	38.5	39.6	36.5
SMS by treatment group (number of patients)				
Positive SMS	57	28	31	54
Negative SMS	418	88	174	332
Indeterminate SMS	49	42	43	48

## Results

The data was analyzed using Microsoft Excel. Sensitivity and specificity were determined for the full data set using pathologic diagnosis of AC. Chi-square tests were performed to determine if non-random associations between categorical variables existed. The Li and Fine method for testing two sensitivities was used for comparing sensitivities and specificities with the Chi-square statistic [12]. Statistical significance was assumed to be *p* < 0.05.

**Fig. 2:**
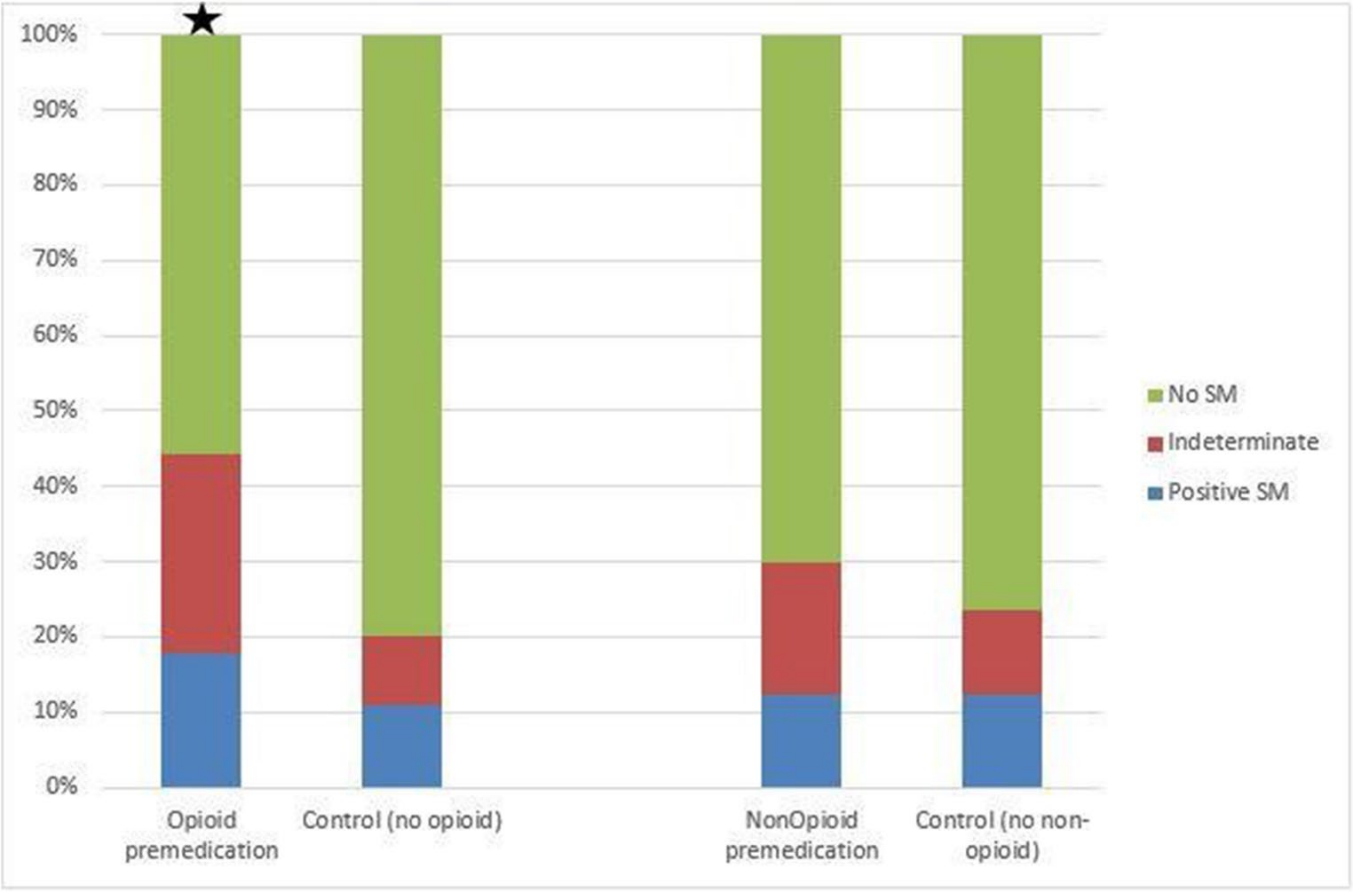
There was a statistically significant increase in the rate of indeterminate SMS in the OA group vs. control (p = < 0.0001 indicated by ★). However, there was no statistically significant difference in the rate of finding an indeterminate SMS in the NOA group vs. control

The null hypothesis that administration of opioid medication prior to gallbladder or right upper quadrant ultrasound does not affect the SMS is rejected (p = < 0.0001). However, NOA administration prior to ultrasound does not have a statistically significant effect on the SMS, and therefore the null hypothesis is accepted for the NOA group (Fig. 2).

Of the 682 cases analyzed, 210 cases had pathology results available for review after cholecystectomy. Of those, 82 specimens had pathology-proven AC, while 128 specimens were given another pathologic diagnosis.

Among the OA group, there was no significant difference in the sensitivity of the SMS in the treatment group as compared to the control group (45.7% vs. 38.3%, respectively; *p* < 0.500). The specificity of the SMS among the treatment group was similar to that of the control group (90.2% vs. 91.8%, respectively; *p* < 0.575).

Among the NOA group, the sensitivity of the SMS was not statistically different among the treatment group when compared to the control group (33.3% vs. 46.2%, respectively; *p* < 0.256). The specificity was similar among the treatment and control groups (90.4% vs. 92.1%, respectively; *p* < 0.452).

**Fig. 3:**
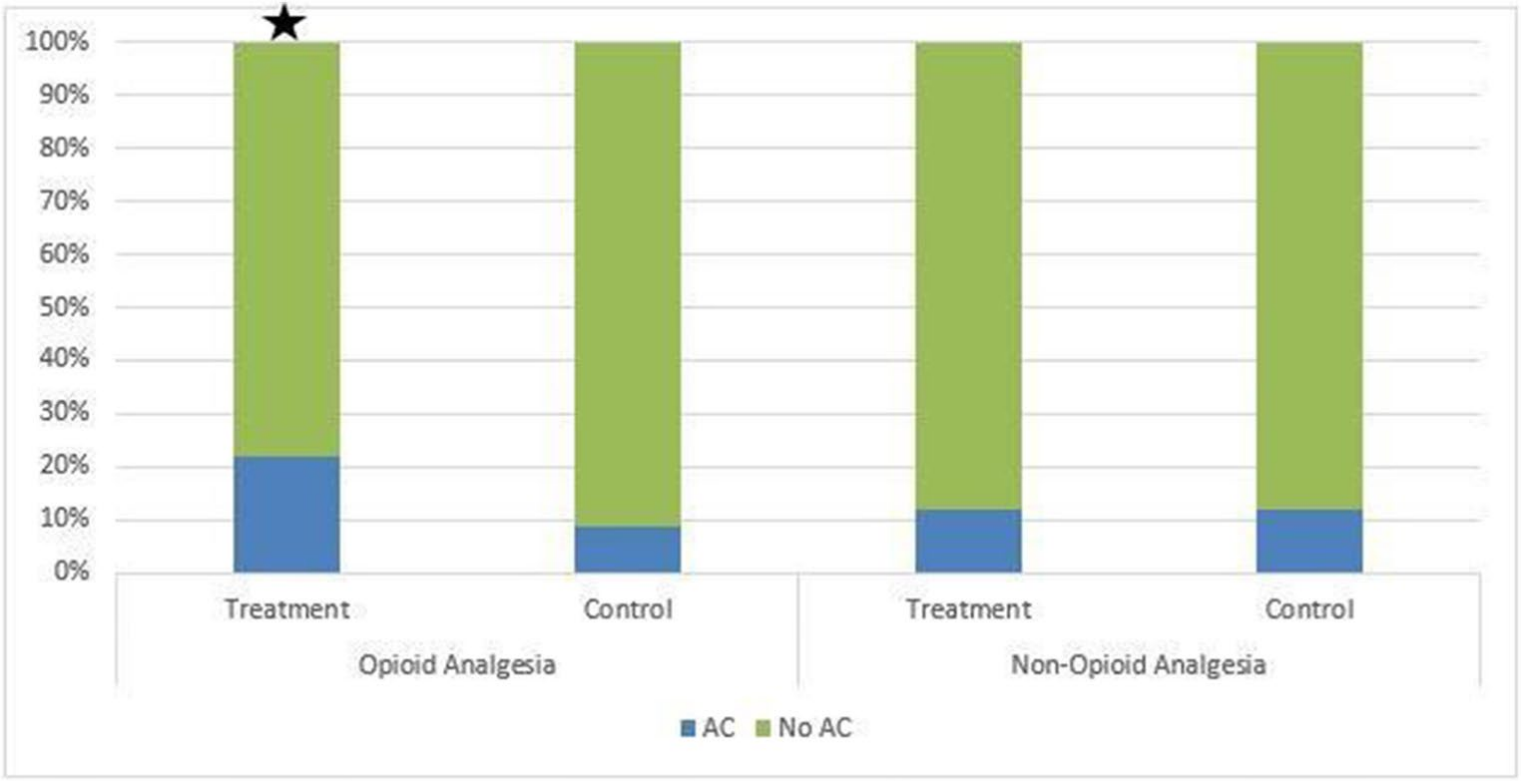
Patients treated with OA were statistically more likely to be diagnosed with AC compared to other treatment groups (*p* < 0.00001 indicated by ★)

There was a statistically significant association between the rate of diagnosis of AC in patients pre-treated with OA versus patients who were not (22.2% vs. 9.0%, respectively; *p* < 0.00001). There was no statistically significant association noted in the NOA group (12.1% vs. 12.0%, respectively; *p* < 0.965). These findings are demonstrated in Fig. 3.

**Fig. 4:**
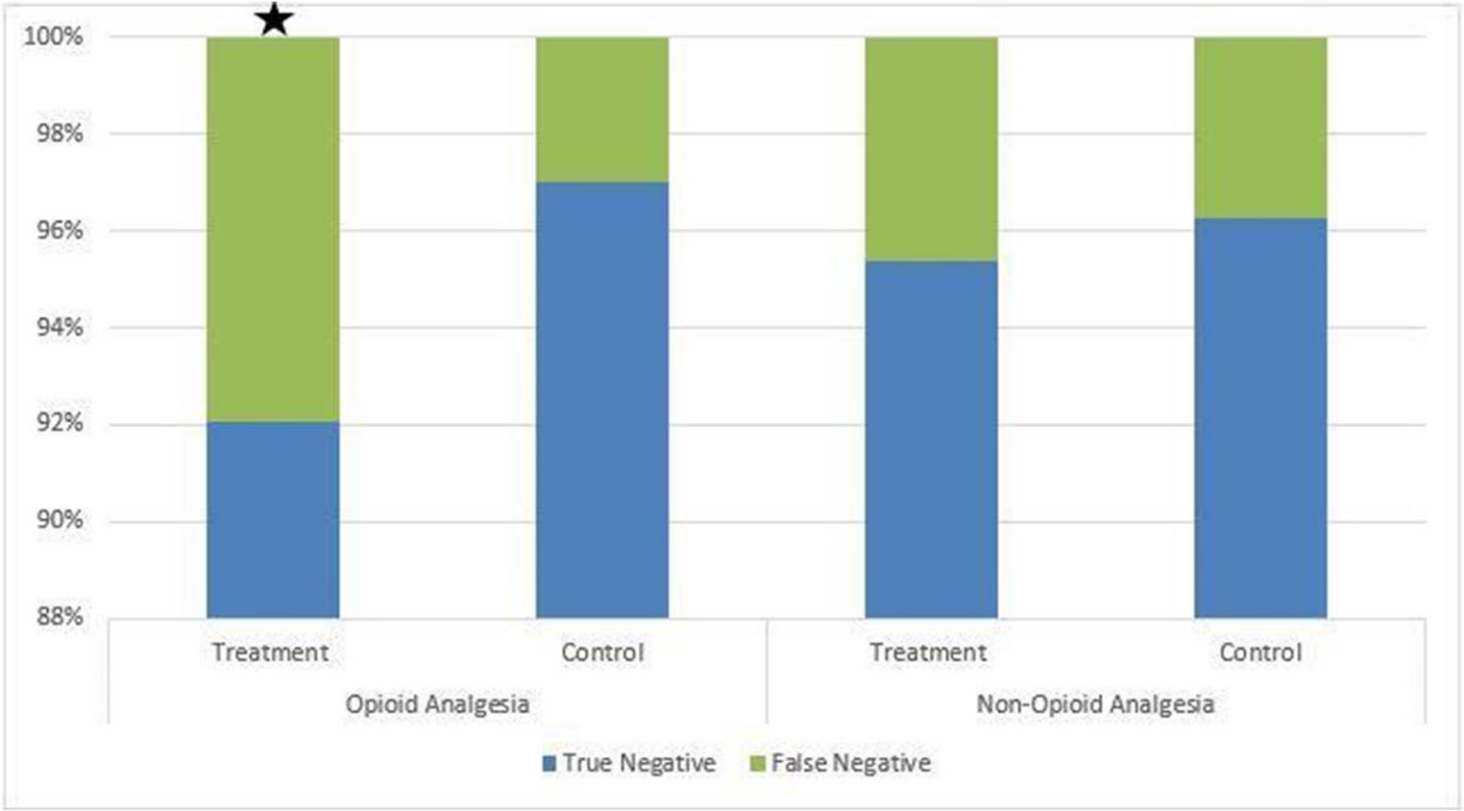
There was a statistically significant increase in the amount of false negative studies in the OA treatment group when compared to other study groups (p<0.013 indicated by ★). This represents 24 cases of missed AC

Patients treated with OA before their ultrasound were statistically more likely to receive a false-negative diagnosis than the control group (7.9% vs. 3.0%, respectively; *p* < 0.013). In other words, the final impression of the radiology report was negative or there was a diagnosis other than AC. On later pathologic review, the correct diagnosis of AC was made. This resulted in 24 cases of missed AC by radiology imaging that were fortunately still identified due to high clinical concern. There was no statistically significant difference in false-negative AC diagnosis between the NOA group compared to the control group (4.6% vs. 3.7%, respectively; *p* < 0.603). These findings are displayed in Fig. 4.

**Fig. 5:**
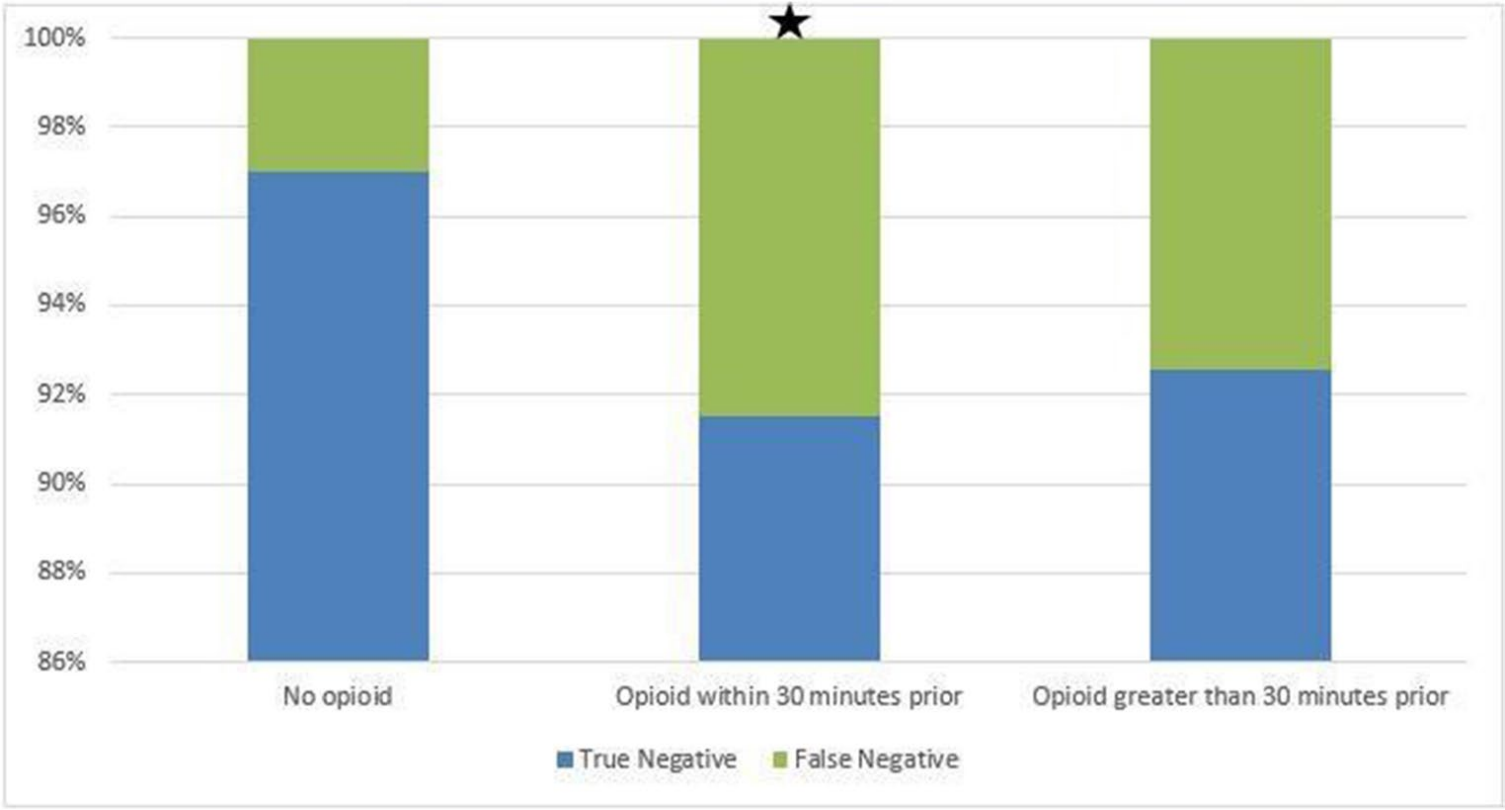
There was a statistically significant increase in the number of false negative ultrasound examinations if opioids were administered within 30 min prior to the examination (p < 0.034 indicated by ★). There was no statistically significant effect after 30 min (p < 0.065)

Time from morphine dose to ultrasound ranged from 0 to 368 min. Patients receiving OA within 30 min of their RUQUS examination were statistically more likely to be given a false-negative diagnosis compared to patients given no OA (8.5% vs. 3.0%, respectively; *p* < 0.034). There was no statistical difference in false-negative results between patients given OA greater than 30 min prior to their ultrasound compared to the control group (7.5% vs. 3.0%, respectively, *p* < 0.065). Please refer to Fig. 5.

Morphine equivalent doses ranged from 2 mg IV to 24 mg IV. Even lower treatment doses (morphine equivalent < 4 mg) were associated with a statistically significant increase in the rate of false-negative results compared to the control group (8.0% vs. 3.0%, respectively; *p* < 0.033).

## Discussion

Two recent studies performed by Noble et al. and Nelson et al. concluded there is likely no relationship between OA and false positive or false negative SMS [13, 14]. There has been criticism of the Noble et al. study, which used small opioid doses, raising questions about the study’s validity. The exact opioid dose given to the patients would have been equianalgesic to only 0.5 or 1.0 mg of IV morphine, depending on whether one or two doses were given according to the study protocol. With such a low dose of opioids, it has been argued that no significant analgesia was given and thus no effect on the SMS could be expected [15]. In our study, morphine equivalent doses ranged from 2 mg IV to 24 mg IV, expanding the generalizability of the results and increasing the likelihood of an effect on the SMS. The Nelson et al. study did not define the doses of opioids administered to patients.

In contrast, several conclusions can be drawn from the results of or study. First, OA resulted in a statistically significant increase in the rate of indeterminate SMS, while NOA administration prior to RUQUS does not affect the rate of finding a positive, negative, or indeterminate SMS. Thus, if a patient presents for RUQUS in the evaluation for AC, premedication with NOA offers the best chance at assessing the SMS accurately.

OA administered prior to RUQUS resulted in a statistically significant increase in false-negative results, including for morphine-equivalent dosing ≤ 4 mg. Therefore, a negative exam in a premedicated patient may yield less reliable results than a non-medicated patient. NOA premedication, however, did not significantly increase false-negative results.

Patients who received OA for treatment of their abdominal pain were statistically more likely to be diagnosed with AC. This is likely a result of increased pain needing a higher level of analgesia than NOA can typically provide. However, there is no statistically significant difference in the sensitivity or specificity of the exam between the opioid treatment group versus the control group.

RUQUS examinations performed within 30 min of premedication with OA resulted in a statistically significant increase in false-negative results. No such effect is seen in the NOA treatment group or in patients treated with OA greater than 30 min prior to scan. This result suggests delaying a RUQUS for at least 30 min after administration of OA can help mitigate the risk of an incorrect diagnosis.

There are several limitations to our study. It was conducted using data from a military treatment facility, where the patient population (patient age, racial distribution, occupational exposures, etc.) may not be completely generalizable to the general public. This may explain why our institutional sensitivity and specificity of positive SMS for AC are lower than published (41.4% for the full sample set, versus 83% published, and 91.5% versus 95% [7]) as well as the increased false-negative results after premedication.

Further, this study does not take into account specific additional sonographic findings (e.g. presence of gallstones, pericholecystic fluid, etc.) other than the SMS that might further contribute to final radiologic diagnosis. There may be a discrepancy between which factors are weighed more heavily by radiologists than by emergency physicians. In a recent survey, 100% of emergency radiologists thought opioids administered prior to the assessment of the SMS would have a negative impact. On the contrary, only 10% of emergency medical physicians surveyed thought opioids would have an adverse effect on the SMS [13]. While it is difficult to quantify retrospectively, presumably the 24 cases of missed AC on radiology imaging received proper medical care due to the emergency physician’s high clinical suspicion based on the patient’s symptoms and/or other secondary imaging findings (gallstones, pericholecystic fluid, wall-thickening, etc.). It is important to consider these differing clinical perspectives when examining the factors contributing to the study results.

## Conclusion

Overall, there is a statistically significant increase in false-negative results after administration of OA. Due to this risk, if a clinician has a high index of suspicion, he or she should consider delaying OA until after the study is completed or delaying the study at least 30 min after the administration of OA. Additionally, our results suggest that administration of NOA in patients under evaluation for possible AC is of little diagnostic consequence and can be a viable alternative treatment option for many patients without sacrificing diagnostic accuracy.

## Data Availability

The data that support the findings of this study are not openly available due to reasons of sensitivity and are available from the corresponding author upon reasonable request.
